# Beyond the supermarket: analyzing household shopping trip patterns that include food at home and away from home retailers

**DOI:** 10.1186/s12889-020-09882-0

**Published:** 2020-11-19

**Authors:** Jackie Yenerall, Wen You, Jennie Hill

**Affiliations:** 1 Department of Agricultural and Resource Economics, University of Tennessee, 2621 Morgan Circle, Knoxville, TN 27996 USA; 2 Department of Public Health Sciences, University of Virginia, 200 Jeanette Lancaster Way, Charlottesville, VA 22903 USA; 3 Department of Epidemiology, University of Nebraska Medical Center, 984395 Nebraska Medical Center, Omaha, NE 68198 USA

**Keywords:** Food shopping behavior, Shopping trip patterns, Food environment

## Abstract

**Background:**

Modifying a household’s food environment by targeting a single retailer type, like supermarkets, has a limited impact on dietary outcomes. This may be because the food environment has a limited impact on shopping behaviors, or because households are not as reliant on supermarkets as we assume. However, our understanding of how households shop for food, especially when considering the use of both food at home (FAH) retailers, such as supermarkets, and away from home retailers (FAFH), such as restaurants, is limited. Thus, understanding how households shop for food is a necessary first step when developing programs to modify food purchasing behavior.

**Methods:**

K-means cluster analysis was used to identify weekly food shopping trip patterns based on the percentage of trips to FAH and FAFH retailers in the 2013 Food Acquisition and Purchase Survey (FoodAPS) dataset (*n* = 4665 households). Multinomial logistic regression was used to examine the relationship between shopping trip patterns, household and food environment characteristics.

**Results:**

Three patterns emerged: primarily supermarket, primarily supercenter, or mix (i.e. no dominant retailer type, but high FAFH use). Households with incomes below 185% of the federal poverty line were evenly divided between patterns that rely primarily on FAH retailers, and the mix pattern. While nearly 70% of households with incomes above 185% of the federal poverty line are in the mix cluster. Supermarket and superstore availability significantly influenced the likelihood of belonging to those clusters respectively, while having a child, higher income, and attitudes towards healthy meal preparation time or taste significantly influenced the likelihood of belonging to the mix cluster.

**Conclusion:**

Although lower-income households are more likely to rely primarily on FAH retailers, household’s, regardless of income, that primarily utilize FAH retailers show a strong preference for either superstores or supermarkets suggesting a need for interventions to reach both retailer types. However, altering the food environment alone may not be sufficient to discourage use of FAFH retailers as households relying on FAFH retailers are significantly influenced by meal preparation time and healthy food taste.

## Background

Where individuals purchase food is believed to influence the quality of their diet and health outcomes [1, 2]. Previous studies investigating this hypothesis have focused primarily on the influence of living within close proximity of or utilizing a specific retailer type (i.e. supermarkets, fast food restaurants), and have produced mixed conclusions regarding the relationship between retailer type and health outcomes [3]. The mixed findings may be the result of the failure to consider the fact that households generally do not rely on a single retailer type to supply food for their entire diet [4, 5]. Failure to consider how and why households utilize multiple retailers may result in policies and programs that emphasize retailer types that may not be relevant to changing consumer behaviors. The consequences of overemphasizing retailer type are exemplified in programs like the Healthy Food Financing Initiative (HFFI). This program subsidizes the cost of building new supermarkets in areas identified as having low access to healthy food options, but evaluations of such interventions have shown little effect on consumer behavior and dietary outcomes [6–8].

The purpose of this paper is to identify household shopping trip patterns in the United States using k-means cluster analysis and examine the household characteristics associated with different patterns. Shopping trip patterns can improve policy makers’ understanding of consumer food shopping behaviors which can inform policy designed to promote healthy food behaviors. By including all food retailers visited during a given time period, this method analyzes how households combine retailers to obtain all purchased food in their diet. K-means cluster analysis sorts households into mutually exclusive groups based on similarities in observed shopping behaviors [9]. The groups identified by the k-means cluster analysis represent the distinct shopping trip patterns that exist in a given population. Understanding how household characteristics influence shopping behavior can help policy makers develop targeted policies that are more relevant to subgroups of consumers, such as low-income families, and may therefore be more effective.

K-means cluster analysis has previously been used to investigate household food shopping trip patterns [4, 5]. Carlson and Kinsey, [4] is the most closely related to the purposes of this article because the data they utilized, the 1994 Continuing Survey of Food Intakes by Individuals (CSFII), includes both food at home (FAH) and food away from home (FAFH) retailers, which include supermarkets and restaurants respectively. While they found that FAFH retailers played an increasingly important role in the second and third most common shopping trip patterns, these findings were based on two-day dietary recalls from a survey collected over two decades ago, therefore more recent data including a longer period of observation is necessary to understand current consumer behaviors [4]. More recently, Stern et al. [5] used cluster analysis to identify shopping patterns using the Nielsen’s National Consumer Panel 2006 and 2012 data. They identified three shopping trip patterns, one primarily utilizing supermarkets, one primarily utilizing mass merchandisers (ex. Walmart), and one that did not rely on a single store type. Although the Stern et al. [5] paper uses more recent data, Nielsen data does not include FAFH, which may bias conclusions regarding shopping trip patterns.

Therefore, this paper will address two needs in the current literature on food shopping trip patterns by using the 2013 Food Acquisition and Purchase Survey (FoodAPS). The first need is the consideration of FAFH retailers in shopping trip pattern analysis. FAFH has become more important to households since the CSFII was collected in 1994 and used in Carlson and Kinsey’s study in 2002. The proportion of household food expenditures and energy intake from FAFH sources have steadily increased since the 1980’s and reached 50 and 34% respectively by 2011 [10]. The FoodAPS survey will allow us to address this gap by identifying more recent shopping trip patterns that include both FAH and FAFH. We will also be able to examine how utilizing only FAH trips may bias our understanding of shopping behavior, by comparing results between analyses using FAH only to combined FAH and FAFH.

The second need is related to understanding the determinants of shopping trip patterns. Previously Carlson and Kinsey, [4] and Stern et al. [5] were able to consider demographic determinants but lacked detailed information on the household food environment, and household attitudes toward healthy food taste, cost, and preparation time. By including an extensive survey of household attributes and perceptions, the FoodAPS survey allows us to estimate the relationship between a broader array of household characteristics, including measures of the food environment, and shopping trip patterns. The results will give some initial insights into potential policies or programs that can more effectively modify consumer shopping behavior.

## Methods

### Data

FoodAPS was administered through the United States Department of Agriculture Economic Research Service (USDA-ERS) from April 2012 to January 2013 to a nationally representative sample of 4826 households [11, 12]. Additionally, the survey oversampled households who participated in the Supplemental Nutrition Program (SNAP) or were low income. SNAP is a food purchasing assistance program for households in the United States with incomes below 130% of the federal poverty line (FPL) [13]. Participating households receive an electronic benefits transaction (EBT) card that can be used to purchase most groceries items (excluding hot-at-the-point-sale goods), and the amount of benefit varies across households based on the household composition, and net income (i.e. household income less some deductions such as the cost of housing). For example, in 2013 the average monthly benefit was $133.07 [14].

The sampling procedure for FoodAPS included four target groups: households participating in SNAP, non-SNAP households with incomes between 100% of the FPL and less than 185% of the FPL, and non-SNAP households with incomes of at least 185% of the FPL [12]. This allowed us to assign households to one of three groups: SNAP participants, low-income non-SNAP participants (non-SNAP households with incomes less than 185% of the FPL), and relatively higher income households (non-SNAP households with incomes greater than 185% of the FPL).

Surveys collected information on all members of the household and identified a primary respondent who was defined as the main food shopper or meal planner. Additional information regarding the administration of the survey can be found using the following link: https://www.ers.usda.gov/data-products/foodaps-national-household-food-acquisition-and-purchase-survey/documentation/. Food expenditure data was collected for seven consecutive days and included food at home (FAH) and food away from home (FAFH). The survey defines FAH as “food and drinks that are brought home and used to prepare meals for consumption at home or elsewhere”, whereas FAFH is defined as “foods and drinks that are obtained and consumed away from home, and prepared foods that are brought home or delivered” [12].

For each shopping trip made by a member of the household during the observation week the survey recorded where the purchase occurred, what was purchased, and how much was spent (i.e. expenditures) [12]. The expenditure data must be interpreted with caution because it can include non-food purchases [15].

For this analysis, only shopping trips that resulted in positive expenditures were included. Thus, shopping trips in which zero expenditures were recorded, or that a household marked as free were excluded. Additionally, any trips that were identified as occurring at unknown or multiple stores types were excluded from the analysis. These two exclusions reduced the sample size to 4665 households.

### Food retailer classification

FoodAPS assigned every retailer to one of 72 place types [15]. These place types were used to construct nine retailer categories used in our analysis: superstore, supermarket, convenience store, grocery store, other food store (ex. farmers market, gas station), restaurant, burger store, other eating place (ex. coffee shop, bar), and other places (ex. casino, work, school). A full list of place types in each retailer category, and a brief description of each category, is available in Table 1.

**Table 1:** Categorization of place types into retail categories

*Retail Category*	*Place Types*	*Description of Category*
Superstore	Superstore	Larger retailers that sell a combination of food and home goods. This category includes retailers like Wal-Mart and Target
Supermarket	Supermarket	Larger food retailer that primarily sells food. This category includes retailers like Kroger, Publix, ShopRite, etc.
Convenience Store	Convenience Store	Small food retailers that generally sell a limited number of products. Includes retailers such as 7–11.
Grocery Store	Combination Grocery/Other; Grocery Store, Large; Grocery Store, Medium; Grocery Store, Not Further Specified	Grocery stores are similar to supermarkets in that they tend to primarily sell food products, but they are much smaller in size.
Other Food Store	Bakery Specialty; Delivery Route; Direct Marketing Farmer; Dollar Store; Farmers Market; Food Bank or Pantry; Fruits and Vegetable Specialty; Gas Station or Market; Liquor Store, Winery; Meat or Poultry Specialty; Nonprofit Food Buying Coop; Pharmacy; Seafood Specialty; Club Stores; Military Commissary; Wholesale	Combines a variety of retailer types that primarily sell food for consumption at home, but are more specialized than supermarket, superstores, grocery and convenience stores.
Restaurant	Buffet Restaurant; Chicken Restaurant; Pizza Restaurant; Restaurant, American; Restaurant, Asian; Restaurant, European; Restaurant, Mexican or Tex-Mex or Latin American; Restaurant, Seafood; Restaurant, Steak House; Restaurant, Not Further Specified	Includes retailers that sell food and meals for consumption away from home. Restaurants likely include retailers for sit-down service and take away.
Burger Restaurant	Burger Restaurant, Including Hot Dog	Includes retailers that sell food and meals for consumption away from home. Burger and hot dog restaurants were included in a separate category because they are more likely than restaurants to be fast-food or fast service restaurants.
Other Eating Place	Bakery; Café and Bakery Café; Coffee Shop; Dairy Desserts; Drinking Place; Miscellaneous Specialty; Sandwich Shop; Travel Place; Vending Machine, Food Truck	Combines a variety of retailer types that primarily sell food for consumption away from home but are more specialized than restaurants.
Other	Athletic Club, Gym; Bowling Alley; Camp, After School Program; Casino; College; Country Club; Fair, Concert, Amusement Park; Family; Fishing or Hunting; Fraternal Organization; Friend; Garden, Home; Garden, Other; Hospital; Institution; Meals on Wheels; Movie Theatre; Municipal Offices; Nonfood Retailer; Park, Community Center; Party, Cookout; Place of Worship; Preschool; School; Work	Combines a variety of retailer types that primarily sell food for consumption away from home but are more specialized and less frequently utilized than restaurants and other eating places.

### Shopping trip patterns

First, for each household the percentage of total shopping trips by retailer category was calculated. Then, k-means cluster analysis was used to sort households into mutually exclusive groups, or clusters, according to similarities in shopping trip patterns, which were defined using the percentage of shopping trips by retailer category [9].

K-means cluster analysis first randomly generates clusters with distinct shopping trip patterns and assigns each household to the cluster with the most similar shopping trip pattern. Then, the average shopping trip pattern is calculated for each cluster. Next, households are re-assigned to the cluster with the cluster-level average shopping trip pattern most similar to their own. The mean for each cluster is then re-calculated. This process of assigning households to a cluster and calculating cluster averages is repeated until no household changes cluster assignment from the last iteration [9].

To assess the importance of FAFH trips in shopping patterns, we used two different definitions of shopping trip patterns in the cluster analysis. First, only FAH shopping trips were included in order to draw comparison with the findings in Stern et al. [5]. Households with no FAH trips (i.e. no expenditures on FAH) were dropped from the analysis, resulting in a sample of 4350 households. Second, all shopping trips during the week (i.e. FAH + FAFH) were included, which resulted in a sample of 4665 households.

Cluster analysis is conducted using a pre-specified number of clusters [9]. For both definitions of shopping trips, a range in the number of clusters from 2 to 5 was considered, which is consistent with the range used in Stern et al. [5]. To facilitate the replicability of the results the random seed generator was set to one. The optimal number of clusters was determined using the pseudo F-statistics, for which higher values indicate more distinct clusters (i.e. larger intra cluster homogeneity, and greater inter cluster heterogeneity) [9].

The FoodAPS data contains household level probability weights to adjust for non-random sampling, unit nonresponse and to make the sample nationally representative [12]. These survey weights were used in our analysis to calculate weighted summary statistics for each cluster based on demographics, socioeconomic status, expenditures, and food environment conditions. Weighted t-tests were used to assess univariate differences across clusters.

### Influence of household characteristics on shopping trip patterns

A weighted multinomial logistic regression (MNL) was used to examine the relationship between shopping trip patterns, represented by the clusters identified in the k-means cluster analysis, and household characteristics. The MNL utilized the same household level probability weights supplied by FoodAPS as used in the summary statsistics. The clusters were the outcome in the MNL and covariates include demographics, household income and time constraints, and taste preferences, which may influence how a household chooses to shop for food [16]. Average marginal effects (AME) are reported and are interpreted as the change in the likelihood of observing a particular shopping trip pattern for a change in a given covariate. Demographics included the self-reported age, sex, race, and education level for the primary respondent and an indicator for living in a rural area. Age was included as a continuous variable. Sex and race were included as binary variables. For sex, a binary variable was created to indicate if the primary respondent identified as female. For race, a binary variable was created to indicate if the primary respondent identified as Caucasian. Education was included in the MNL as a continuous variable, but because it is a categorical variable (i.e. the education variable measured the highest level of education received, not the years spent in school) it should not be interpreted directly but was included to control for the effect of education. FoodAPS used the definition of rural from the ERS Food Access Research Atlas, which is based on census tract population. Thus, the binary variable for living in a rural area indicated if a household lived in a census tract that had a population of fewer than 2500 people [17].

Income constraint variables included the household’s monthly income and an indicator for belief it costs too much to eat healthy. Income was a continuous variable that measured a household’s monthly income in U.S. dollars. In the survey the primary respondent was asked if it costs too much to eat healthy foods [17]. Responses to this question were used to create a binary variable that captured the belief that it costs too much to eat healthy.

Time constraint variables captured the availability of time to shop or prepare meals. Variables included a binary indicator for the presence of a child (defined as a household member who was 18 years of age or younger), the percentage of adults in the household who work (defined as the percentage of all adults in the household who reported being employed in the previous week), a binary indicator for access to a vehicle (defined as leasing or owning a vehicle), binary indicator if the primary respondent reported that they did not have adequate time to prepare healthy food.

Finally, food environment variables were included to capture the availability of superstores, supermarkets, fast food restaurants, and non-fast food restaurants. Availability was defined as the count of food retailers in a specific radius of the household and to align with the USDA definition of access a radius of 1 mile was used for urban households and 10 miles for rural households [18].

Finally, taste preferences variables included a binary indicator for region of residence (Northeast, Midwest, South, West) and a binary indicator if the primary respondent reported that they did not believe healthy food tastes good. FoodAPS includes an indicator for the household’s U.S. Census region [17]. Regional indicators capture the heterogeneity in food cultures that exist across the United States and the potential for variations in availability of food based on factors such as seasonality and/or cultural norms and preferences.

All analysis was conducted in Stata, version 13.

## Results

### K-means clustering analysis

**Fig. 1:**
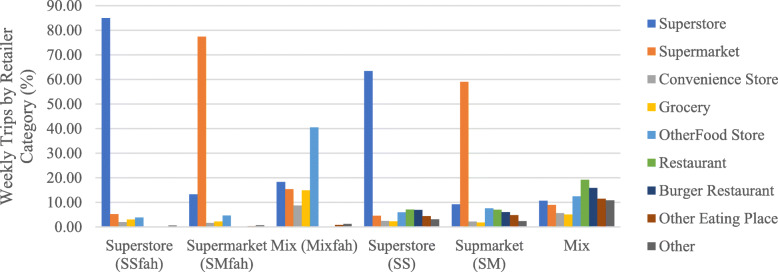
Shopping Trip Patterns (Clusters) by Retailer Category for FAH Only or FAH and FAFH Analysis. Mix = Cluster in which no single retailer type dominated shopping trip patterns. FAH = food at home. FAFH = food away from home

Analyses using either FAH or FAFH+FAFH trips identified three clusters that were named after the predominant retailer category: superstore (SS), supermarket (SM), and mix (M) (see Fig. 1). In both analyses, the mix cluster did not have a single dominant retailer category. Thus, the mix name reflects the use of a variety of retailers.

For ease of discussion, those clusters from the FAH trips only cluster analysis have a FAH superscript on the name (i.e. SS^fah^ indicates the superstore cluster from the analysis using FAH only shopping trips). There are no superscripts on the clusters from FAH + FAFH trips. Additionally, because the FoodAPS dataset defines FAH events by “food and drinks that are brought home and used to prepare meals for consumption at home or elsewhere”, rather than store type, it is possible that a trip to a store type typically associated with FAFH, such as a restaurant, may be included in FAH only analysis.

As indicated by the cluster name, households in either the SS^fah^ or SM^fah^ clusters relied primarily on a single retailer for their weekly trips (i.e. superstores and supermarkets respectively), which accounted for 85.00 and 77.37% of their average weekly trips respectively. On the other hand, households in the SS and SM clusters were comparatively less reliant on their dominant store type which accounted for 63.39 and 59.06% of weekly trips respectively. This indicates that even households who relied primarily upon a single store type incorporated FAFH into their weekly food supply. For households in the mix^fah^ cluster the other food store category was the most frequently utilized, accounting for approximately 40.48% of weekly trips. While in the mix cluster, restaurants were the most frequented retailer, accounting for about 19.20% of weekly trips.

**Fig. 2:**
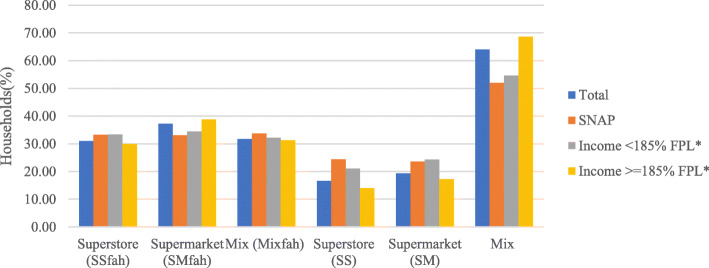
Distribution of Households Across Shopping Trip Patterns by Income and SNAP Participation. fah suffix indicates results of cluster analysis that analyzed food at home (FAH) trips only. Mix = Cluster in which no single retailer type dominated shopping trip patterns. SNAP = Supplemental Nutrition Assistance Program. FPL = Federal poverty line. *excludes SNAP Participants

The most striking difference between the FAH only and FAH + FAFH analyses was the distribution of households across the clusters, which resulted in very different cluster sizes between the two analyses (see Fig. 2). Considering FAH trips only, households were fairly evenly distributed across all three clusters. The SM^fah^ cluster was the largest with 37.29% of households, and SS^fah^ the smallest with 31.74% of households. This distribution was consistent within each of the income subgroups: SNAP participants, SNAP non-participants with incomes of less than 185% of the federal poverty line (FPL), and SNAP non-participants with incomes of at least 185% of the FPL. However, SNAP non-participants with incomes of at least 185% of the FPL has a slightly larger SM^fah^ cluster as compared to the lower income groups.

Including FAFH trips in the analysis resulted in a significant shift in the distribution of the households. Now a majority, 63.99%, belonged to the mix cluster, as compared to 31.74% in the mix^fah^. SM was the next largest with 19.36% of households, and SS the smallest with 16.64% of households. This pattern was consistent within all income subgroups except the SNAP subgroup, where the SS cluster was just slightly larger than the SM cluster with 24.40 and 23.61% of households respectively.

This analysis shows that ignoring FAFH sources will result in an inaccurate representation of how most households shop for their weekly food supply. Thus, the remainder of this paper only utilizes the results from the cluster analysis for FAH + FAFH trips.

### Household characteristics for FAH + FAFH clusters

To compare household characteristics across clusters we used two t-test to test for significant differences across households based on similar shopping patterns. First, we tested for differences between the primarily FAH clusters (SS vs SM). Then the primarily FAH clusters were combined (SS + SM) and tested for differences with the primarily FAFH mix cluster (SS + SM vs mix). For the remainder of this section, when discussing the results of the t-tests we will use SS vs SM and SS + SM vs mix to refer to t-test comparing between the primarily FAH clusters, or between the primarily FAH and FAFH clusters. Otherwise, we will refer to the descriptive statistics for each cluster using the cluster’s name.

In general, the greatest number of significant differences occurred when comparing the SS and SM to the mix cluster (i.e. SM + SS vs mix) (Table 2). The mix cluster had the largest household size on average (mean = 2.6, Linearized Square Error [LSE] = 0.06) as compared to the other clusters (SM + SS vs mix pval< 0.001), and was more likely to have a child present (mean = 38.04%, LSE = 1.94) as compared to the other clusters (SM + SS vs mix pval< 0.001).

**Table 2:** Household Socioeconomic and food environment characteristics by food shopping trip patterns clusters

Mean (LSE)	Total Population	Superstore (SS)	Supermarket (SM)	Mix	Weighted t-tests	
					SS vs SM	SS + SM vs Mix
*Household Characteristics*						
Size	2.45 (0.05)	2.30 (0.09)	2.11 (0.07)	2.60 (0.06)		***
Presence of Child (%)	33.20 (1.37)	28.08 (2.44)	21.64 (1.81)	38.04 (1.94)	*	***
Monthly Income	5194.19 (206.38)	4238.03 (287.38)	4524.11 (252.49)	5645.63 (260.25)		***
SNAP Participant (%)	13.19 (0.88)	19.34 (1.64)	16.09 (1.90)	10.72 (1.00)		***
Working Adults (%)	58.68 (1.03)	52.00 (3.33)	46.61 (2.21)	64.07 (1.25)		***
Vehicle Access (%)	89.52 (1.20)	88.97 (2.34)	87.28 (1.78)	90.35 (1.26)		
*Healthy Food Attitudes*						
Not enough time to prepare healthy meals *(%)*	20.23 (0.74)	15.94 (2.03)	12.83 (1.89)	23.57 (1.12)		***
Healthy Food too Costly *(%)*	32.43 (1.44)	34.07 (3.38)	31.15 (2.88)	32.39 (1.96)		
Healthy Food Does Not Taste Good *(%)*	21.70 (1.02)	19.41 (2.29)	13.59 (1.34)	24.74 (1.39)	*	***
*Food Environment*						
Superstore Availability	2.04 (0.29)	1.99 (0.23)	1.76 (0.35)	2.14 (0.36)		
Supermarket Availability	2.60 (0.50)	1.70 (0.28)	2.84 (0.43)	2.77 (0.63)	**	
Fast Food Restaurant Availability	12.52 (1.56)	9.91 (1.40)	12.47 (2.30)	13.22 (1.77)		
Non-Fast Food Restaurant Availability	55.33 (7.74)	41.33 (6.94)	56.46 (10.24)	58.62 (8.71)		
*Regional Indicator*						
Rural (%)	34.34 (3.67)	39.05 (4.52)	28.42 (4.80)	34.91 (4.15)		
Northeast (%)	15.61 (2.47)	8.93 (2.87)	16.20 (3.47)	17.17 (2.63)		**
Midwest (%)	31.15 (3.52)	46.19 (8.06)	15.71 (5.11)	31.91 (3.59)	*	
South (%)	35.22 (3.99)	31.78 (6.53)	43.35 (6.49)	33.66 (4.09)		
West (%)	18.01 (2.71)	13.09 (3.31)	24.74 (5.08)	17.26 (2.54)	*	

Means of all variables are reported. The means of binary variables are reported as percentages (%). Household income is measured as the monthly income in U.S. dollars. Working adults measured the percentages of all adults in the household that are currently working. Vehicle access indicates that a household owns or leases a vehicle. FoodAPS used the definition of rural from the ERS Food Access Research Atlas, which is based on census tract population. Thus, the binary variable for living in a rural area indicates if a household lived in a census tract that had a population of fewer than 2500 people. Food environment variables measure the count of a specific retailer type within 1-mile radius of households in urban areas, and 10-mile radius of households in rural areas

*Mix* Cluster in which no single retailer type dominated shopping trip patterns

*SNAP* Supplemental Nutrition Assistance Program

*LSE* linearized standard errors

* = *p* < 0.05 ** = *p* < 0.01 *** = *p* < 0.001

Households in the mix cluster were also wealthier than the SS or SM cluster. Mix households have a higher monthly household income (mean = $5645.63, LSE = 260.25, SM + SS vs mix pval< 0.001) and lower SNAP participation (mean = 10.72%, LSE = 1) (SM + SS vs mix pval< 0.001). This difference may in part be driven by the greater percentage of adults that were working in the mix households (mean = 64.07%, LSE = 1.25) as compared to the other clusters (SM + SS vs mix pval< 0.001).

There were also significant differences across clusters based on healthy food attitudes. Households in the mix cluster were most likely to report they were too busy to prepare healthy foods (mean = 23.57%, LSE = 1.12) (SM + SS vs mix pval< 0.001). Finally, households in the mix cluster were most likely to report that healthy food does not taste good (mean = 24.75%, LSE = 1.39), followed by the SS cluster (mean = 19.41%, LSE = 2.29) and SM cluster (mean = 13.59%, LSE = 1.34). This difference was significant when comparing between the SS and SM clusters (pval = 0.018) or between the primarily FAH and FAFH clusters (SM + SS vs mix pval< 0.001).

Households in the mix cluster were more likely to live in the northeast (mean = 17.17%, LSE = 2.63), as compared to households in the SM (mean = 16.20%, LSE = 3.47) or SS cluster (mean = 8.93%, LSE = 2.87) (SM + SS vs mix pval = 0.009). Households in the SS cluster were more likely to live in the Midwest (mean = 46.19%, LSE = 8.06) as compared to households in SM cluster (mean = 15.71%, LSE = 5.11) (SM vs SS pval = 0.012). Households in the SM cluster were more likely to live in the west (mean = 24.74%, LSE = 5.08) as compared to households in SS cluster (mean = 13.09%, LSE = 3.31) (SM vs SS pval = 0.015).

There were far fewer differences between the clusters when considering the household’s food environment (see Table 2). The only food environment variable that was significantly different between the clusters was supermarket availability. As might be expected, supermarket availability was greater amongst households in the SM cluster.

(mean = 2.84, LSE = 0.43), as compared to SS cluster (mean = 1.70, LSE = 0.28) (SM vs SS pval = 0.004)

Demographic information for the primary respondent is available in Table 3. The primary respondent for the mix cluster tended to be younger as compared to the SS or SM cluster. Approximately 75% of primary respondents for the mix cluster were below the age of 59 as compared to at most 60% in either the SS or SM cluster. Primary respondents in the SS cluster were the most likely to have at most a high school degree (mean = 30.83%, LSE = 2.7), while primary respondents in the mix cluster were most likely to have at least a college degree (mean = 35.87%, LSE = 2.64).

**Table 3:** Primary respondent characteristics by shopping trip pattern (cluster)

Mean (LSE)	Total Population	Superstore (SS)	Supermarket (SM)	Mix	Weighted t-tests	
					SS vs SM	SS + SM vs Mix
Female *(%)*	68.68 (1.10)	66.53 (2.38)	68.35 (3.78)	69.34 (1.48)		
Married *(%)*	46.23 (1.60)	41.77 (2.63)	39.97 (2.93)	49.28 (1.87)		**
*Age*						
Less than 35*(%)*	22.99 (1.22)	21.50 (1.41)	14.87 (2.40)	25.82 (1.76)	*	**
36–59*(%)*	45.94 (1.53)	39.34 (2.70)	38.96 (3.33)	49.75 (1.69)		***
Over 60*(%)*	31.07 (1.42)	39.16 (3.09)	46.17 (3.97)	24.43 (1.73)		***
*Race*						
African American *(%)*	11.22 (1.48)	9.99 (2.61)	10.28 (1.94)	11.82 (1.59)		
Caucasian *(%)*	77.63 (1.90)	80.02 (3.75)	78.96 (2.66)	76.60 (2.05)		
Other *(%)*	11.15 (1.17)	9.99 (1.98)	10.76 (1.58)	11.58 (1.29)		
*Education*						
Less Than High School *(%)*	9.29 (1.05)	12.58 (2.44)	11.96 (1.75)	7.64 (1.03)		**
High School *(%)*	24.12 (1.44)	30.83 (2.70)	24.13 (2.00)	22.38 (1.76)	*	*
Some College or Associates Degree *(%)*	33.27 (1.79)	33.13 (3.48)	30.59 (3.36)	34.12 (2.00)		
College and More *(%)*	33.31 (2.11)	23.46 (2.87)	33.32 (3.55)	35.87 (2.64)	*	*

Means of all variables are reported. The means of binary variables are reported as percentages (%). Primary respondents’ sex, marital status, race, and education are all self-reported. Education measures the highest level of education received

*Mix* Cluster in which no single retailer type dominated shopping trip patterns

*LSE* Linearized standard errors

* = *p* < 0.05 ** = *p* < 0.01 *** = *p* < 0.001

Trip frequency and expenditure comparisons are available in Table 4. Households in the mix cluster averaged the greatest number of trips per week (mean = 10.01, LSE = 0.16) due to their frequent trips to FAFH retailers (mean = 6.82, LSE = 0.17), which was on average three times greater than either the SS or SM cluster. Households in the mix cluster also had the highest expenditures for the week (mean = $191.65, LSE = 5.33), while households in the SS and SM clusters had very similar weekly expenditures at $139.19(LSE = 7.81) and $140.30(LSE = 7.01) respectively.

**Table 4:** Trip counts and expenditures by shopping trip patterns (cluster)

Mean (LSE)	Total Population	Superstore (SS)	Supermarket (SM)	Mix	Weighted t-tests	
					SS vs SM	SS + SM vs Mix
*Trip Counts*						
Total	8.34(0.14)	5.32(0.26)	5.41 (0.23)	10.01 (0.16)		
FAH	3.28 (0.05)	3.36 (0.14)	3.54 (0.13)	3.19 (0.06)		
FAFH	5.05 (0.14)	1.96 (0.15)	1.87 (0.12)	6.82 (0.17)		
*Trip Count Percentages*						
FAH (%)	49.84 (0.86)	72.03 (1.32)	72.96 (1.16)	37.08 (0.76)		
FAFH (%)	50.16 (0.86)	27.97 (1.32)	27.04 (1.16)	62.92 (0.76)		
*Expenditures*						
Total	173.06 (3.91)	139.19 (7.81)	140.30 (7.01)	191.65 (5.33)		***
FAH (%)	68.80 (0.60)	86.17 (0.80)	82.98 (1.18)	60.06 (0.71)	*	***
FAFH (%)	31.20 (0.60)	13.83 (0.80)	17.02 (1.18)	39.94 (0.71)	*	***

Expenditures are measured for a week and reported in U.S. dollars

*Mix* Cluster in which no single retailer type dominated shopping trip patterns

*FAH* Food at home

*FAFH* Food away from home

*LSE* Linearized standard errors

* = p < 0.05 ** = p < 0.01 *** = p < 0.001

### Influence of household characteristics on shopping trip patterns

Average marginal effects (AME), which used the SM cluster as the base case, were reported from the MNL to determine the relationship between different household characteristics and the likelihood of belonging to a given shopping trip pattern represented by the clusters (Table 5). Covariates included in the regression but not reported in Table 5 include access to a vehicle, rural indicator, and primary respondent age, sex, race, self-reported education. Full results are available in Additional File 1. Increasing a household’s monthly income increased the probability of belonging to the mix cluster (AME = 0.1012, pval = 0.02) but decreased the probability of belonging to the SM cluster (AME = -0.0519, pval = 0.02).

**Table 5:** Influence of Household Characteristics on Likelihood of Belonging to Different Shopping Trip Patterns (Clusters)

AME (LSE)	Superstore (SS) (LSE)	Supermarket (SM) (LSE)	Mix(M) (LSE)
*Income Constraint Variables*			
Monthly Income (in $10,000)	−0.0494 (0.0378)	− 0.0519* (0.0218)	0.1012* (0.0426)
Healthy Food too Costly *(%)*	0.0110 (0.0249)	−0.0003 (0.0169)	−0.0107 (0.0298)
*Time Constraint Variables*			
Percentage of Adults Working	−0.0001 (0.0002)	−0.0004 (0.0002)	0.0004 (0.0003)
Presence of Child	−0.113 (0.0195)	−0.0407* (0.0172)	0.0520* (0.0247)
Not Enough Time to Prepare Healthy Meals	−0.062 (0.0233)	−0.0688* (0.0269)	0.1050*** (0.0264)
*Food Environment Variables*			
Superstore Availability	0.0297*** (0.0065)	−0.0252** (0.0079)	−0.0044 (0.0094)
Supermarket Availability	−0.0235** (0.0088)	0.0160** (0.0054)	0.0075 (0.0070)
Fast-Food Restaurant Availability	−0.0015 (0.0009)	0.0006 (0.0010)	0.0010 (0.0012)
Restaurant Availability	0.0001 (0.0002)	0.00004 (0.0002)	−0.0002 (0.0002)
*Taste Preference Variables*			
Healthy Food Does Not Taste Good	−0.0280 (0.0272)	−0.0769*** (0.0191)	0.1049** (0.0305)
Midwest	0.1352** (0.0487)	−0.1068 (0.0568)	−0.0284 (0.0364)
South	0.0884* (0.0366)	0.0291 (0.0401)	−0.1175** (0.0385)
West	0.0397 (0.0378)	0.0592 (0.0310)	−0.0989* (0.0470)

Covariates included in the regression but not reported include access to a vehicle, rural indicator, and primary respondent age, sex, race, self-reported education. Full results are available in Additional file 1. Household income is measured as the monthly income in U.S. dollars. Percentage of working adults measured the percentages of all adults in the household that are currently working. Food environment variables measured the count of a specific retailer type within 1-mile radius of households in urban areas, and 10-mile radius of households in rural areas. Binary variables that measure attitudes towards healthy food (healthy food too costly, not enough time to prepare healthy meals, healthy food does not taste good) are measured using the primary respondents’ affirmative responses to these statements

*Mix* Cluster in which no single retailer type dominated shopping trip patterns

*AME* Average marginal effect

*LSE* Linearized standard error

* = p < 0.05 ** = p < 0.01 *** = p < 0.001

Time constraints also influenced a household’s likelihood of belonging to different clusters. The presence of a child significantly increased the probability of belonging to the mix cluster (AME = 0.0520, pval = 0.04) but decreased the probability of belonging to the SM cluster (AME = -0.0407, pval = 0.02). Finally, belief that there is not enough time to prepare healthy meals increased the probability of belonging to the mix cluster (AME = 0.1050, pval< 0.001) but decreased the probability of choosing the SM cluster (AME = -0.0688, pval = 0.01).

Availability of superstores and supermarkets had the expected sign and influence for the SS and SM clusters and no significant effect for the mix cluster. The availability of superstores increased the probability of belonging to the SS cluster (AME = 0.0297, pval< 0.001) but decreased the probability of belonging to the SM cluster (AME = -0.0252, pval = 0.001). The availability of supermarkets decreased the probability of belonging to the SS cluster (AME = -0.0235, pval = 0.007) but increased the probability of belonging to the SM cluster (AME = 0.016, pval = 0.003).

Finally, tastes preferences also had a significant influence. If households indicated that healthy food does not taste good it increased the probability of belonging to the mix cluster (AME = 0.1049, pval = 0.001) but decreased the probability of belonging to the SM cluster (AME = -0.0769, pval< 0.001). Regional indicators also suggested that the living in the south decreased the probability of belonging to the mix cluster (AME = -0.1175, pval = 0.002) but increased the probability of belonging to the SS cluster (AME = 0.0884, pval = 0.016). Living in the Midwest also increased the probability of belonging the SS cluster (AME = 0.1352, pval = 0.006).

## Discussion

This paper used a dataset that is nationally representative for the United States, the 2013 Food Acquisition and Purchase Survey (FoodAPS), to identify weekly shopping trip patterns based on the percentage of trips by retailer category using k-means cluster analysis. In order to assess the importance of FAFH, we conducted two cluster analyses, one using only FAH shopping trips and one using both FAH and FAFH shopping trips. Using the clusters that included FAH and FAFH trips, a multinomial regression was used to estimate the relationship between household level characteristics and associated shopping trip patterns. The results provided three important insights for policy makers to consider.

First, FAFH was an important source of food for households. This was demonstrated by the dramatic shift in the distribution of households across shopping trip patterns after including FAFH trips. Utilizing only FAH trips we found that households were fairly evenly distributed across three clusters: superstore (SS^fah^), supermarket (SM^fah^), and mix^fah^. However, after including FAFH trips 64% of households were in the mix cluster as compared to 31.74% in mix^fah^. This is significant because households in the mix cluster utilized FAFH for a majority of their weekly trips. This finding demonstrates the importance of FAFH sources in a majority of households’ weekly food shopping trips and suggests that utilizing data limited to FAH sources may not accurately capture typical shopping patterns. Thus, interventions, programs or policies that focus solely on FAH may have a limited impact as they may not target the retailors that are most relevant to the consumers.

Second, we found that households primarily utilizing a single retailer during the week had strong preferences for either supermarkets or superstores. This finding is similar to Stern et al. [5], who also identified two shopping trip patterns that primarily utilized supermarkets or superstores. Additionally, we found that households in the superstore cluster were more likely to have a child present in the home, as compared to the supermarket cluster, and even after controlling for the availability of superstores and supermarkets in our MNL model, were more likely to live in the Midwest or the South.

Prior retailer-based policies and interventions have emphasized supermarkets because many households regularly utilize them for FAH shopping, and they are believed to an important source of healthy foods [6, 19]. While superstores have received less attention, previous research has shown that the entry of a new Walmart, a type of superstore, may lower food insecurity rates, and there is some evidence that household purchases at superstores are lower in nutritional quality than purchases from supermarkets [1, 20]. Our research provides further evidence that superstores should be considered when designing policies to promote healthy food behaviors among low-income households, especially those living in the South and Midwest.

Third, analyzing household characteristics associated with each shopping pattern may be useful for developing interventions that target specific subpopulations, such as households with children. Our results found that households with children are most likely to belong to the mix cluster. Given previous research that found food purchased from FAFH retailers tended to be lower in nutritional quality it is concerning that households with children are most likely to be in the mix cluster because this shopping trip pattern frequently utilizes FAFH retailers [21–23]. Although the investigation between shopping trip patterns and dietary quality is beyond the scope of this paper, we believe it will be important for future research to investigate the influence of shopping trip patterns on dietary outcomes in order to provide direct insights into healthy eating promotion policy designs.

Households in the mix cluster also had a higher percentage of working adults and we found that for this cluster their choice of where to shop was sensitive to the time it takes to prepare a meal and belief healthy food does not taste good. Thus, interventions designed to change the availability of a certain retailer may not influence this group’s behavior if the interventions do not also address their preferences and time constraints.

Our results are consistent with previous research that found most households, even those with strong preferences towards superstores or supermarkets, also utilized a wide variety of additional retailer types [4, 5]. Our research adds to the limited body of research that suggests policy makers may need to address how and why households utilize multiple retailers to develop more effective interventions that target retailers that are relevant to consumer behavior, rather than targeting retailers they assume are important [5]. Past retailer-based policies have focused on single retailer type, whether it is building new supermarkets through the Healthy Food Financing Initiative (HFFI), regulating the location of fast food restaurants or modifying the quality of food available at convenience stores [19, 24]. Thus, future research should focus on determining how shopping trip patterns influence a household’s choice of food purchases and the dietary quality of purchases in order to identify effective policy instruments to promote healthy eating.

### Study limitations

Our findings should be used with the following limitations in mind. First, there are no standard methods for classifying food retailers, which limits our results’ comparability with other studies. Second, cluster analysis is a data driven method and as such can be sensitive to choices in shopping behavior that is used to identify clusters, or the classification of food retailers.

Additionally, this study excluded all shopping trips that resulted in zero expenditures or were identified as free by the respondents. This excluded those trips to food pantries, soup kitchens, and free school lunches. Although the excluded households are only a small fraction of the data (3%), any behaviors specific to the utilization of free food and meals would not be captured in this analysis and is beyond the scope of this paper. Future research should investigate how household incorporate free food and meals into their food supply especially for those low-income subpopulations.

Finally, the results from our regression analysis only describe correlations between household characteristics and shopping trip patterns and cannot be used to describe any causal relationships.

## Conclusion

By classifying households into mutually exclusive groups based on the similarities in their weekly food shopping patterns our study provides three important insights for policy makers in the United States interested in developing programs to alter consumer food shopping behavior.

First, relying on the results from shopping trip pattern studies using only FAH may overestimate the importance of superstores and supermarkets in weekly shopping trips. By comparing the results from two cluster analysis (one including FAH only and another include FAH + FAFH) we found a significant shift in the distribution of households away from superstore and supermarket utilization towards FAFH retailers, as represented by the increased prevalence of households in the mix cluster. This pattern was consistent across all income subgroups and suggests the need to further investigate and address the determinants of multiple store utilization rather than focusing on policies that increase the availability of any single store type.

Second, after including FAFH trips we found that households that still primarily relied on FAH retailers for their weekly food shopping trips had strong preferences for either superstore or supermarkets, and that choice of either shopping pattern was influenced by the availability of superstores and supermarkets in their local food environment. This suggests policy makers may need to be mindful of local food environment conditions when developing programs to target shoppers who primarily use food at home retailers, and they may need to target superstores, which are generally not the focus on shopping interventions.

Finally, we found that approximately 64% of households in our sample made a majority of their weekly food shopping trips to FAFH retailers, as captured by the mix cluster. This is particularly concerning because FAFH retailers tend to offer food lower in nutritional quality and households in the mix cluster were more likely to have a child present. Targeting these families may require interventions that address their attitudes related to healthy food as the choice of the mix cluster was not influenced by the local food environment but was sensitive to the time it takes to prepare a meal and belief healthy food does not taste good.

## Supplementary Information

**Additional file 1.** Influence of Household Characteristics and Food Environment on Likelihood of Belonging to Different Shopping Trip Patterns (Clusters). Additional file 1 and contains the full results for the regression reported in Table 5. This includes the coefficients for the variable’s vehicle, rural indicator, and primary respondent age, sex, race, self-reported education, which were included in the original analysis but not reported in Table 5.

## Data Availability

This study utilized the Public-Use data from the Food Acquisition and Purchase Survey (Food-APS). This data can be accessed using the following link: https://www.ers.usda.gov/data-products/foodaps-national-household-food-acquisition-and-purchase-survey/ The citation for the data is: Economic Research Service (ERS), U.S. Department of Agriculture (USDA). National Household Food Acquisition and Purchase Survey (FoodAPS). 2013. https://www.ers.usda.gov/foodaps and it is included in the references.

## Abbreviations

FAFH: Food Away From Home
FAH: Food at Home
FoodAPS: Food Acquisition and Purchase Survey
HFFI: Healthy Food Financing Initiative
CSFII: Continuing Survey of Food Intakes by Individuals
USDA-ERS: United States Department of Agriculture Economic Research Service
SNAP: Supplemental Nutrition Program
FPL: Federal Poverty Line
MNL: Multinomial Logistic Regression
AME: Average Marginal Effects
SS: Superstore Cluster
SM: Supermarket Cluster
M: Mix Cluster
