# Supplementary material for: Beyond the supermarket: analyzing household shopping trip patterns that include food at home and away from home retailers
Source: BMC Public Health. 2020 Nov 19;20:1747. doi: 10.1186/s12889-020-09882-0 (PMC7678129; doi:10.1186/s12889-020-09882-0)
Supplement: Supplementary file 1 — Additional file 1. Influence of Household Characteristics and Food Environment on Likelihood of Belonging to Different Shopping Trip Patterns (Clusters). Additional file 1 and contains the full results for the regression reported in Table 5. This includes the coefficients for the variable’s vehicle, rural indicator, and primary respondent age, sex, race, self-reported education, which were included in the original analysis but not reported in Table 5. [file 12889_2020_9882_MOESM1_ESM.docx]

| **Additional File 1**  Influence of Household Characteristics and Food Environment on Likelihood of Belonging to Different Shopping Trip Patterns (Clusters) | | | |
| --- | --- | --- | --- |
|  | Superstore(SS)  (SE) | Supermarket(SM)  (SE) | Mix(M)  (SE) |
| *Income Constraint Variables* | | | |
| Monthly income (in $10,000) | -0.0494  (0.0378) | -0.0519*  (0.0218) | 0.1012* (0.0426) |
| Healthy Food too Costly *(%)* | 0.0110  (0.0249) | -0.0003  (0.0169) | -0.0107  (0.0298) |
| *Time Constraint Variables* | | | |
| Percentage of Adults Working^a^ | -0.0001  (0.0002) | -0.0004  (0.0002) | 0.0004  (0.0003) |
| Presence of Child ^a^ | -0.113  (0.0195) | -0.0407*  (0.0172) | 0.0520* (0.0247) |
| Not Enough Time to Prepare Healthy Meals | -0.062  (0.0233) | -0.0688*  (0.0269) | 0.1050***  (0.0264) |
| *Food Environment Variables* | | | |
| Superstore Availability ^a^ | 0.0297***  (0.0065) | -0.0252**  (0.0079) | -0.0044  (0.0094) |
| Supermarket Availability | -0.0235**  (0.0088) | 0.0160**  (0.0054) | 0.0075  (0.0070) |
| Fast-Food Restaurant  Availability | -0.0015  (0.0009) | 0.0006  (0.0010) | 0.0010  (0.0012) |
| Restaurant Availability | 0.0001  (0.0002) | 0.00004  (0.0002) | -0.0002  (0.0002) |
| *Taste Preference Variables* | | | |
| Healthy Food Does Not Taste Good | -0.0280  (0.0272) | -0.0769***  (0.0191) | 0.1049**  (0.0305) |
| Midwest | 0.1352**  (0.0487) | -0.1068  (0.0568) | -0.0284  (0.0364) |
| South | 0.0884*  (0.0366) | 0.0291  (0.0401) | -0.1175**  (0.0385) |
| West | 0.0397  (0.0378) | 0.0592  (0.0310) | -0.0989*  (0.0470) |
| Rural | 0.0119  (0.0181) | -0.0611  (0.0369) | 0.0492  (0.0341) |
| *Household Characteristics and Primary Respondent Demographics* | | | |
| Access to vehicle | 0.0272  (0.0269) | -0.0177  (0.0247) | -0.0095  (0.0364) |
| Age | 0.0008  (0.0006) | 0.0024**  (0.0008) | -0.0033**  (0.0010) |
| Female | -0.0156  (0.0162) | 0.0016  (0.0287) | 0.0140  (0.0299) |
| Caucasian | 0.0060  (0.0240) | 0.0164  (0.0171) | -0.0223  (0.0267) |
| Education | -0.0229*  (0.0096) | -0.0031  (0.0070) | 0.0260*  (0.0111) |
| Values represent the average marginal effects (AME) with linearized standard errors in parenthesis, using the supermarket (SM) cluster as the base case, from a weighted multinomial logistic regression using the clusters as the outcome. Education is a self-reported education categorial variable and is not indented to be interpreted directly  **=p<0.05 **=p<0.01 ***=p<0.001* | | | |
